# Pathologic response after preoperative therapy predicts prognosis of Chinese colorectal cancer patients with liver metastases

**DOI:** 10.1186/s40880-017-0244-1

**Published:** 2017-10-02

**Authors:** Yun Wang, Yun-Fei Yuan, Hao-Cheng Lin, Bin-Kui Li, Feng-Hua Wang, Zhi-Qiang Wang, Pei-Rong Ding, Gong Chen, Xiao-Jun Wu, Zhen-Hai Lu, Zhi-Zhong Pan, De-Sen Wan, Peng Sun, Shu-Mei Yan, Rui-Hua Xu, Yu-Hong Li

**Affiliations:** 1 Sun Yat-sen University Cancer Center, State Key Laboratory of Oncology in South China, Collaborative Innovation Center for Cancer Medicine, Guangzhou, 510060 Guangdong P. R. China; 2 Department of Medical Oncology, Sun Yat-sen University Cancer Center, Guangzhou, 510060 Guangdong P. R. China; 3 Department of Hepatobiliary Surgery, Sun Yat-sen University Cancer Center, Guangzhou, 510060 Guangdong P. R. China; 4 Department of Colorectal Surgery, Sun Yat-sen University Cancer Center, Guangzhou, 510060 Guangdong P. R. China; 5 Department of Pathology, Sun Yat-sen University Cancer Center, Guangzhou, 510060 Guangdong P. R. China

**Keywords:** Colorectal cancer, Liver metastases, Chemotherapy, Pathologic response

## Abstract

**Background:**

Pathologic response is evaluated according to the extent of tumor regression and is used to estimate the efficacy of preoperative treatment. Several studies have reported the association between the pathologic response and clinical outcomes of colorectal cancer patients with liver metastases who underwent hepatectomy. However, to date, no data from Chinese patients have been reported. In this study, we aimed to evaluate the association between the pathologic response to pre-hepatectomy chemotherapy and prognosis in a cohort of Chinese patients.

**Patients and methods:**

In this retrospective study, we analyzed the data of 380 liver metastases in 159 patients. The pathologic response was evaluated according to the tumor regression grade (TRG). The prognostic role of pathologic response in recurrence-free survival (RFS) and overall survival (OS) was assessed using Kaplan–Meier curves with the log-rank test and multivariate Cox models. Factors that had potential influence on pathologic response were also analyzed using multivariate logistic regression and Kruskal–Wallis/Mann–Whitney *U* tests.

**Results:**

Patients whose tumors achieved pathologic response after preoperative chemotherapy had significant longer RFS and OS than patients whose tumor had no pathologic response to chemotherapy (median RFS: 9.9 vs. 6.5 months, *P* = 0.009; median OS: 40.7 vs. 28.1 months, *P* = 0.040). Multivariate logistic regression and Kruskal–Wallis/Mann–Whitney *U* tests showed that metastases with small diameter, metastases from the left-side primary tumors, and metastases from patients receiving long-duration chemotherapy had higher pathologic response rates than their control metastases (all *P* < 0.05). A decrease in the serum carcinoembryonic antigen (CEA) level after preoperative chemotherapy predicted an increased pathologic response rate (*P* < 0.05). Although the application of targeted therapy did not significantly influence TRG scores of all cases of metastases, the addition of cetuximab to chemotherapy resulted in a higher pathologic response rate when combined with irinotecan-based regimens rather than with oxaliplatin-based regimens.

**Conclusions:**

We found that the evaluation of pathologic response may predict the prognosis of Chinese colorectal cancer patients with liver metastases after preoperative chemotherapy. Small tumor diameter, long-duration chemotherapy, left primary tumor, and decreased serum CEA level after chemotherapy are associated with increased pathologic response rates.

## Introduction

Colorectal cancer is the fifth most common cancer and the leading cause of cancer-related death in China, with an increasing incidence of 4%–6% annually [1–4]. The liver is the most common organ for colorectal cancer metastases, and liver metastasis is the main cause of death of patients with colorectal cancer [5, 6]. Currently, surgical resection is the primary therapeutic strategy for patients with isolated liver metastases; it results in an improved clinical outcome, with a 5-year overall survival (OS) rate of 58% [7]. The administration of chemotherapy before surgical resection has been shown to increase not only the curative resection rate for initially unresectable lesions but also the disease-free survival rate for patients with resectable disease [8–10]. Thus, preoperative chemotherapy followed by hepatectomy is recommended for colorectal cancer patients with liver metastases, especially for patients at high risk [9, 11–13].

Currently, the efficacy of preoperative chemotherapy is often evaluated based on the radiologic response, according to Response Evaluation Criteria in Solid Tumors (RECIST) [14], using computed tomography or magnetic resonance imaging (MRI). However, prolonged survival is not always associated with radiologic response, especially when targeted therapy agents are added to the chemotherapy regimen [15]. Pathologic response is another evaluation index that could be used to evaluate the efficacy of preoperative chemotherapy. The pathologic response of colorectal cancer patients with liver metastases is generally evaluated according to the tumor regression grade (TRG) [16]. Rubbia-Brandt et al. [16] reported an association between the pathologic response and OS in colorectal cancer patients with resected liver metastases, which was confirmed by Blazer et al. [17], who suggested that the pathologic response might be used as a new outcome endpoint after surgical resection of liver colorectal metastases. Additionally, the pathologic response is also regarded as a predictive factor for survival in patients with other metastatic diseases, including metastatic breast cancer [18], gastric cancer [19], esophageal cancer [20, 21], and pancreatic cancer [22], who receive preoperative chemotherapy or radiotherapy. Several studies have attempted to determine factors that might be associated with pathologic response, such as the duration of preoperative chemotherapy and the combination of bevacizumab and preoperative traditional chemotherapy [23–26]. However, the number of cases in these studies was relatively small, and to date no data obtained in studies of Chinese patients have been reported.

In this retrospective study, we analyzed the metastatic lesions that were resected from colorectal cancer patients with liver metastases. We aimed to demonstrate the association between the pathologic response to pre-hepatectomy chemotherapy and prognosis in colorectal cancer patients with liver metastases. We also analyzed factors that might have a potential effect on TRG.

## Patients and methods

### Patients

This retrospective study was performed at Sun Yat-sen University Cancer Center in Guangzhou, China. All patients who were consecutively diagnosed with colorectal cancer and liver metastases and therefore underwent hepatectomy after preoperative chemotherapy between June 2002 and December 2015 were included. Inclusion criteria were as follows: (1) pathologic diagnosis of colorectal cancer with liver metastases; (2) preoperative fluorouracil-based chemotherapy as a first/second-line treatment; and (3) adequate metastasis specimens for analysis. Patients with extrahepatic metastases and those who underwent a prior hepatectomy were excluded. This study was approved by the Ethical Review Board of Sun Yat-sen University Cancer Center. Informed consent was waived given the study’s non-interventional retrospective design. The authenticity of this article was validated by uploading the key raw data to the Research Data Deposit public platform (http://www.researchdata.org.cn), with Approval Number RDDA2017000150.

### Pathologic response assessment

**Fig. 1:**
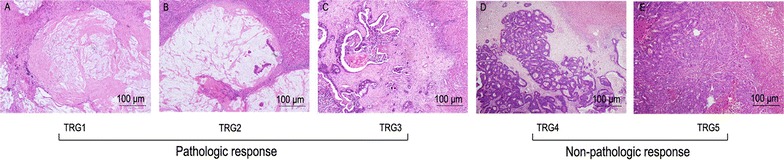
Pathologic response according to the tumor regression grade (TRG) scoring standard. **A** TRG1, absence of residual cancer cells and an abundance of fibrosis. **B** TRG2, a large amount of fibrosis and scattered rare residual cancer cells. **C** TRG3, more residual cancer cells, but fibrosis predominates. **D** TRG4, presence of abundant residual cancer cells that predominate over fibrosis; and **E** TRG5, almost completely cancer cells with an absence of fibrosis

Two independent pathologists evaluated the pathologic response of metastatic colorectal lesions according to hematoxylin- and eosin-stained slides. Pathologic response was scored according to the TRG, as suggested by Rubbia-Brandt et al. [16], based on the proportion of residual cancer cells and fibrosis in the metastatic lesions (Fig. 1). The pathologic responses of patients with multiple metastases were evaluated at two different analyses. To determine patient outcomes, such as recurrence-free survival (RFS) and OS, we conducted patient-related analyses. We used the highest TRG (i.e., the worst grade) among all metastases in one patient to be the pathologic response of that patient. To analyze the TRG-influenced factors, the tumor-related analyses were used. Specifically, we considered all the TRG scores evaluated in each liver metastatic tumor. Moreover, pathologic responses were classified into three levels according to the TRG scores of metastases: TRG1–2 indicated a major pathologic response, TRG3 indicated a partial pathologic response, and TRG4–5 indicated no pathologic response. Finally, metastases with TRG1–3 were classified in the pathologic response group, whereas metastases with TRG4–5 were classified in the non-pathologic response group.

### Follow-up

Follow-up was performed until August 2016. Dates of relapse and death were verified by hospital records or by phone contact with the patients or their relatives. Radiologic responses were evaluated by computed tomograph or MRI according to RECIST version 1.1 [14]. The responses were then classified as complete response (CR), partial response (PR), stable disease (SD), or progressive disease (PD) [14]. Other clinicopathologic information that was collected from patient records included age, sex, primary tumor location, and level of preoperative carcinoembryonic antigen (CEA). The TNM stage of the tumors was reclassified according to the Union for International Cancer Control TNM Staging System (7th edition). Metastases were grouped into small-size tumor (diameter < 2.5 cm) and large-size tumor (diameter ≥ 2.5 cm). Short duration of preoperative chemotherapy was defined as ≤ 4 cycles for 2-week regimens or ≤ 3 cycles for 3-week regimens, whereas long duration of preoperative chemotherapy was defined as > 4 cycles for 2-week regimens or > 3 cycles for 3-week regimens.

### Statistical analysis

Kaplan–Meier curves with log-rank tests were used to compare the RFS and OS rates in different TRG levels. RFS was defined as the date of hepatectomy to the date of the first relapse at any site or death from any cause without relapse; the patients who were lost during follow-up or still alive or relapse-free at the last follow-up were censored. OS was calculated from hepatectomy to death from any cause; the patients who were lost during follow-up or still alive at the last follow-up were censored. A multivariate logistic regression was performed as a preliminary screen for potential factors associated with TRG. Kruskal–Wallis or Mann–Whitney *U* tests were used to further compare the pathologic response rates between different parameters, including tumor size, duration of preoperative chemotherapy, preoperative CEA level, primary tumor site, and different combinations of chemotherapy regimens. All the statistical analyses were performed using SPSS software version 22.0 (Chicago, IL, USA) with two-tailed tests. A *P* value of < 0.05 was considered statistically significance.

## Results

### Patient characteristics

A total of 159 colorectal cancer patients with 380 liver metastatic lesions were included in the present study. Patient characteristics are shown in Table 1. All 159 patients received fluorouracil-based preoperative chemotherapy regimens as a first-line or second-line treatment. Of the 159 patients, 114 (71.7%) received preoperative chemotherapy without targeted therapy (non-targeted therapy group); 22 (13.8%) received chemotherapy in combination with bevacizumab (bevacizumab-combined therapy group); and 23 (14.5%) received chemotherapy in combination with cetuximab (cetuximab-combined therapy group). The median ages of the patients in the non-targeted therapy group, bevacizumab-combined therapy group, and cetuximab-combined therapy group were 52 years (range 35–83 years), 43 years (range 32–71 years), and 59 years (range 28–76 years), respectively. For all patients treated with cetuximab, the status of *Kras* was confirmed as wild-type before cetuximab administration. Seventy-eight (57.4%) of 136 patients in the non-targeted therapy and bevacizumab-combined therapy groups underwent the *Kras* examination, with a *Kras* mutation rate of 38.5% (30 of 78; 23 patients in the non-targeted therapy group and 7 in bevacizumab-combined therapy group). The patient characteristics based on liver metastases are shown in Table 2. Of the 380 metastatic lesions that were resected and evaluated, 292 were classified into the pathologic response group (TRG1–3, 76.8%), and 88 were classified into the non-pathologic response group (TRG4–5, 23.2%).

**Table 1:** Clinicopathologic characteristics of colorectal cancer patients with liver metastases according to preoperative chemotherapy

Variable	Non-targeted therapy (*n* = 114)	Bev-combined therapy (*n* = 22)	Cet-combined therapy (*n* = 23)
Sex			
Man	72 (63.2)	14 (63.6)	15 (65.2)
Woman	42 (36.8)	8 (36.4)	8 (34.8)
Primary tumor site			
Right side	18 (15.8)	7 (31.8)	4 (17.4)
Left side	96 (84.2)	15 (68.2)	19 (82.6)
Primary tumor grade			
G1–2	84 (73.7)	18 (81.8)	16 (69.6)
G3	30 (26.3)	4 (18.2)	7 (30.4)
Histological subtype			
Non-mucinous	102 (89.5)	20 (90.9)	20 (87.0)
Mucinous	12 (10.5)	2 (9.1)	3 (13.0)
Primary tumor pT category^a^			
pT1–2	11 (9.6)	1 (4.5)	2 (8.7)
pT3–4	103 (90.4)	21 (95.5)	21 (91.3)
Primary tumor pN category^a^			
pN0	57 (50.0)	12 (54.5)	8 (34.8)
pN1-2	57 (50.0)	10 (45.5)	15 (65.2)
Preoperative CEA level			
≤ 5 ng/mL	45 (39.5)	7 (31.8)	9 (39.1)
> 5 ng/mL	69 (60.5)	15 (68.2)	14 (60.9)
Metastasis presentation			
Synchronous	92 (80.7)	18 (81.8)	16 (69.6)
Metachronous	22 (19.3)	4 (18.2)	7 (30.4)
Number of metastases per patient^b^	3 (1–32)	3 (1–14)	6 (1–15)
Resection status of liver metastases			
R0	88 (77.2)	16 (72.7)	15 (65.2)
R1–2	26 (22.8)	6 (27.2)	8 (34.7)
Cycles of preoperative chemotherapy^b^	4 (2–12)	6 (3–11)	4 (2–8)
Duration of preoperative chemotherapy^c^			
Short duration	58 (50.9)	8 (36.4)	11 (47.8)
Long duration	56 (49.1)	14 (63.6)	12 (52.2)
Chemotherapy backbone			
Oxa-based	83 (72.8)	13 (59.1)	5 (21.7)
Iri-based	26 (22.8)	9 (40.9)	17 (73.9)
Oxa + Iri-based	3 (2.6)	0 (0.0)	1 (4.3)
Fluoropyrimidine only	2 (1.8)	0 (0.0)	0 (0.0)
Postoperative chemotherapy			
Yes	70 (61.4)	15 (68.2)	14 (60.9)
No	44 (38.6)	7 (31.8)	9 (39.1)
Response^d^			
PR	39 (36.4)	14 (63.6)	17 (73.9)
SD	47 (43.9)	8 (36.4)	6 (26.1)
PD	21 (19.6)	0 (0.0)	0 (0.0)
TRG^e^			
1–2	32 (28.1)	8 (36.4)	4 (17.4)
3	39 (34.2)	7 (31.8)	10 (43.5)
4–5	43 (37.7)	7 (31.8)	9 (39.1)

*Bev* bevacizumab, *Cet* cetuximab, *CEA* carcinoembryonic antigen, *Oxa* oxaliplatin, *Iri* irinotecan, *PR* partial response, *SD* stable disease, *PD* progression disease, *TRG* tumor regression grade

^a^ According to the Union for International Cancer Control (UICC) staging system (7th edition)

^b^ These data are presented as median followed by range in parentheses; others are presented as number of patients followed by percentages in parentheses

^c^ Short duration of preoperative chemotherapy: ≤ 4 cycles for 2-week regimens or ≤ 3 cycles for 3-week regimens; long duration of preoperative chemotherapy: > 4 cycles for 2-week regimens or > 3 cycles for 3-week regimens

^d^ Because the initial computed tomography or magnetic resonance imaging was performed in other hospitals, 7 of 114 patients who received preoperative chemotherapy without targeted therapy were not evaluable for radiologic response

^e^ TRG was evaluated based on the patient-related analysis

**Table 2:** Clinicopathologic characteristics of patients based on liver metastatic tumors

Characteristic	Non-targeted therapy	Bev-combined therapy	Cet-combined therapy
Metastatic tumors	252 (66.3)	44 (11.6)	84 (22.1)
Primary tumor site			
Right side	36 (14.3)	17 (38.6)	14 (16.7)
Left side	216 (85.7)	27 (61.4)	70 (83.3)
Primary tumor grade			
G1–2	189 (75.0)	35 (79.5)	50 (59.5)
G3	63 (25.0)	9 (20.5)	34 (40.5)
Histological subtype of primary tumor			
Non-mucinous	228 (90.5)	42 (95.5)	74 (88.1)
Mucinous	24 (9.5)	2 (4.5)	10 (11.9)
Primary tumor pT category^a^			
pT1–2	31 (12.3)	2 (4.5)	4 (4.8)
pT3–4	221 (87.7)	42 (95.5)	80 (95.2)
Primary tumor pN category^a^			
pN0	106 (42.1)	29 (65.9)	26 (31.0)
pN1–2	146 (57.9)	15 (34.1)	58 (69.0)
CEA decline^b^			
Yes	136 (54.0)	35 (79.5)	55 (65.5)
No	56 (22.2)	7 (15.9)	6 (7.1)
Unevaluable	60 (23.8)	2 (4.5)	23 (27.4)
Metastases presentation			
Synchronous	208 (82.5)	40 (90.9)	67 (79.8)
Metachronous	44 (17.5)	4 (9.1)	17 (20.2)
Size of metastases (cm)			
< 2.5	154 (61.1)	15 (34.1)	58 (69.0)
≥ 2.5	98 (38.9)	29 (65.9)	26 (31.0)
Resection status of liver metastases			
R0	171 (67.9)	36 (81.8)	48 (57.1)
R1–2	81 (32.1)	8 (18.2)	36 (42.9)
Duration of preoperative chemotherapy^c^			
Short duration	109 (43.3)	23 (52.3)	31 (36.9)
Long duration	143 (56.7)	21 (47.7)	53 (63.1)
Chemotherapy backbone			
Oxa-based	184 (73.0)	29 (65.9)	16 (19.0)
Iri-based	52 (20.6)	15 (34.1)	66 (78.6)
Oxa + Iri-based	13 (5.2)	0 (0.0)	2 (2.4)
Fluoropyrimidine only	3 (1.2)	0 (0.0)	0 (0.0)
TRG^d^			
1–2	95 (37.7)	21 (47.7)	32 (38.1)
3	97 (38.5)	15 (34.1)	32 (38.1)
4–5	60 (23.8)	8 (18.2)	20 (23.8)

All data are presented as number of metastases followed by percentages in parentheses

*Bev* bevacizumab, *Cet* cetuximab, *CEA* carcinoembryonic antigen, *Oxa* oxaliplatin, *Iri* irinotecan, *TRG* tumor regression grade

^a^ According to the Union for International Cancer Control (UICC) staging system (7th edition)

^b^ The CEA decline was based on the comparison between the preoperative CEA and the baseline CEA detection. Because initial CEA levels were unavailable, 30 of 159 patients (85 of 380 available tumors) were not evaluable for CEA variations

^c^ Short duration of preoperative chemotherapy: ≤ 4 cycles for 2-week regimens or ≤ 3 cycles for 3-week regimens; long duration of preoperative chemotherapy: > 4 cycles for 2-week regimens or > 3 cycles for 3-week regimens

^d^ TRG was evaluated as metastases-related analysis

### Survival predictive value of the pathologic response

**Fig. 2:**
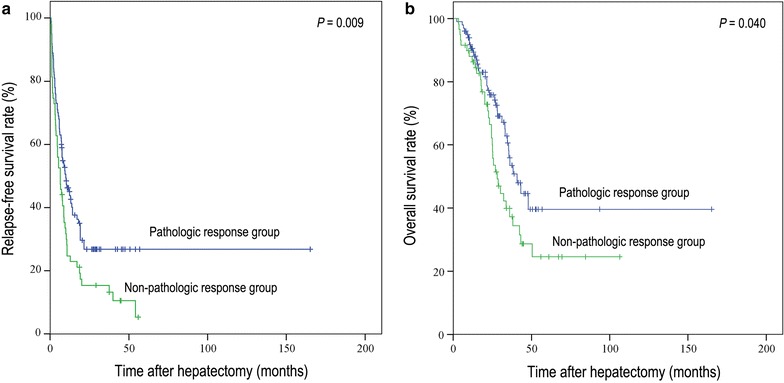
Kaplan–Meier curves of relapse-free survival (**a**) and overall survival (**b**) in colorectal cancer patients with liver metastases after hepatectomy based on pathologic response. Patients in the pathologic response group had a longer recurrence-free survival (RFS) (median RFS: 9.9 vs. 6.5 months, *P* = 0.009; **a**) and longer overall survival (OS) (median OS: 40.7 vs. 28.1 months, *P* = 0.040; **b**) than patients in the non-pathologic response group

Results were based on the patient-related TRG analysis. Patients in the pathologic response group presented with a better RFS than patients in the non-pathologic response group (median RFS, 9.9 vs. 6.5 months; 3-year RFS rate, 26.8% vs. 15.4%, *P* = 0.009; Fig. 2a). Consistently, patients in the pathologic response group also had a better OS than patients in the non-pathologic response group (median OS, 40.7 vs. 28.1 months; 5-year OS rate, 39.6% vs. 24.6%, *P* = 0.040; Fig. 2b). After adjusting for other clinicopathologic factors in the multivariate Cox analysis, patients in the non-pathologic response group were estimated to have a 71.0% RFS disadvantage (hazard ratio [HR], 1.71, 95% confidence interval [CI], 1.15–2.56) and a 71.0% OS disadvantage (HR, 1.71, 95% CI 1.02–2.88) compared with patients in the pathologic response group, respectively (Table 3).

**Table 3:** Predictive factors for survival by multivariate Cox analysis

Variable	Relapse-free survival		Overall survival	
	HR (95% CI)	*P* value^d^	HR (95% CI)	*P* value^d^
Age (> 65 vs. ≤ 65 years)	1.22 (0.68–2.18)	0.508	1.78 (0.86–3.72)	0.123
Primary tumor site (left vs. right)	1.36 (0.81–2.29)	0.244	0.75 (0.39–1.43)	0.382
Tumor grade (G3 vs. G1–2)	0.99 (0.79–1.24)	0.937	0.85 (0.63–1.15)	0.290
pT category (pT3–4 vs. pT1–2)	1.07 (0.55–2.10)	0.843	1.28 (0.47–3.49)	0.624
pN category (pN1–2 vs. pN0)	1.06 (0.70–1.61)	0.775	1.16 (0.66–2.04)	0.617
Preoperative CEA level (> 5 vs. ≤ 5 ng/mL)	0.83 (0.55–1.25)	0.380	1.43 (0.81–2.50)	0.216
Interval from primary tumor resection to liver metastases (> 12 vs. ≤ 12 months)	0.96 (0.55–1.68)	0.881	1.18 (0.58–2.40)	0.649
Number of metastases (> 1 vs. 1 per patient)	0.96 (0.58–1.60)	0.883	0.87 (0.44–1.75)	0.703
Maximum size of metastases (> 5 vs. ≤ 5 cm)	0.82 (0.48–1.41)	0.482	1.48 (0.81–2.71)	0.199
Resection status (R1–2 vs. R0)	2.27 (1.45–3.56)	*<* *0.001*	2.00 (1.13–3.54)	*0.017*
Radiologic response (SD/PD vs. PR)^a^	0.75 (0.49–1.15)	0.184	1.23 (0.67–2.25)	0.497
Duration of preoperative chemotherapy (long vs. short)^b^	1.28 (0.86–1.91)	0.231	0.81 (0.46–1.41)	0.451
Chemotherapy backbone (CT with Bev vs. CT only)	1.33 (0.74–2.39)	0.340	1.33 (0.61–2.92)	0.472
Chemotherapy backbone (CT with Cet vs. CT only)	1.24 (0.70–2.22)	0.458	1.68 (0.82–3.42)	0.153
Postoperative chemotherapy (yes vs. no)	0.50 (0.33–0.76)	*0.001*	0.50 (0.28–0.90)	*0.021*
TRG (TRG4–5 vs. TRG1–3)^c^	1.71 (1.15–2.56)	*0.008*	1.71 (1.02–2.88)	*0.041*

*Bev* bevacizumab, *Cet* cetuximab, *CEA* carcinoembryonic antigen, *CT* chemotherapy, *PR* partial response, *SD* stable disease, *PD* progressive disease, *TRG* tumor regression grade

^a^ Because the initial computed tomography or magnetic resonance imaging was performed in other hospitals, 7 of 114 patients who received preoperative chemotherapy without targeted therapies were not evaluable for radiologic response

^b^ Short duration of preoperative chemotherapy: ≤ 4 cycles for 2-week regimens or ≤ 3 cycles for 3-week regimens; long duration of preoperative chemotherapy: > 4 cycles for 2-week regimens or > 3 cycles for 3-week regimens

^c^ TRG was evaluated based on the patient-related analysis

^d^ *P* < 0.05 was emphasized in italics

### Factors and their influence on the TRG

Based on the tumor-related analysis, we included the factors into the binary logistic regression model of TRG and found that small tumor size (odds ratio [OR], 0.50; 95% CI 0.27–0.92; *P* = 0.025), long-duration of chemotherapy (OR, 0.28; 95% CI 0.14–0.53; *P* < 0.001), primary tumor at left side (OR, 0.44; 95% CI 0.21–0.92; *P* = 0.030), and a decline in the CEA level (OR, 0.48; 95% CI 0.24–0.94; *P* = 0.032) after preoperative chemotherapy were favorable predictors of TRG (Table 4).

**Table 4:** Multivariable logistic analyses of potential benefit factors for pathologic response in terms of tumor-related analysis

Variable	OR (95% CI)	*P* value^b^
Age (> 65 vs. ≤ 65 years)	0.84 (0.30–2.35)	0.743
Primary tumor site (left vs. right)	0.44 (0.21–0.92)	*0.030*
pN category (pN1-2 vs. pN0)	1.04 (0.56–1.91)	0.903
CEA decline (yes vs. no)^a^	0.48 (0.24–0.94)	*0.032*
Metastases presentation (metachronous vs. synchronous)	0.72 (0.30–1.74)	0.462
Size of metastases (< 2.5 vs. ≥ 2.5 cm)	0.50 (0.27–0.92)	*0.025*
Resection status (R1–2 vs. R0)	1.09 (0.56–2.13)	0.794
Preoperative chemotherapy backbone (CT with Bev vs. CT only)	0.29 (0.10–0.83)	*0.021*
Preoperative chemotherapy backbone (CT with Cet vs. CT only)	1.79 (0.86–3.73)	0.120
Duration of preoperative chemotherapy (long vs. short)	0.28 (0.14–0.53)	*<* *0.001*

*CEA* carcinoembryonic antigen, *CT* chemotherapy, *Bev* bevacizumab, *Cet* cetuximab

^a^ Because initial CEA levels were unavailable, 30 of 159 patients (85 of 380 available tumors) were not evaluable for CEA variations

^b^ *P* < 0.05 was emphasized in italics

**Fig. 3:**
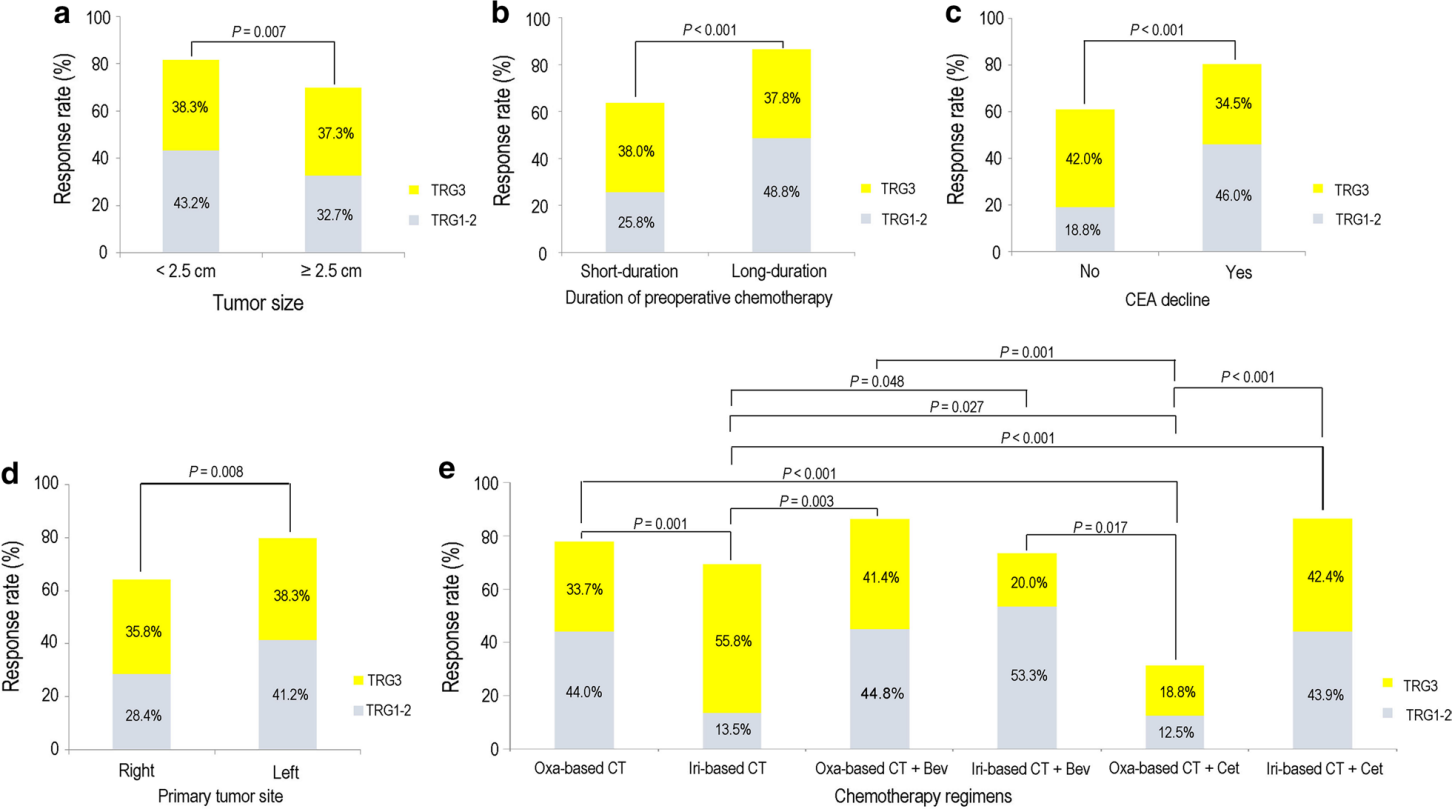
Pathologic response of colorectal cancer patients with liver metastasis according to potential factors. Metastases were classified according to tumor size (**a**), duration of preoperative chemotherapy (**b**), CEA variation (**c**), primary tumor site (**d**), and preoperative treatment (**e**). The distribution (%) of partial histologic response (TRG3) and major histologic response (TRG1–2) statuses was reported within each category of tumor size, duration of preoperative chemotherapy, CEA variation, primary tumor site and preoperative treatment. For preoperative treatment, the comparisons were conducted between every two regimens. Only significant pairwise comparisons were noted (*P* < 0.05). *Oxa* oxaliplatin, *Iri* irinotecan, *Bev* bevacizumab, *Cet* cetuximab

In all metastases, 227 (59.7%) were categorized as small tumors; of these, 185 achieved a pathologic response. Whereas, 153 metastases (40.3%) were categorized as large tumors; of these, 107 achieved a pathologic response. Compared with large tumors, small tumors had increased pathologic response rate (TRG1–2 43.2% and TRG3 38.3% vs. TRG1–2 32.7% and TRG3 37.3%, *P* = 0.007; Fig. 3a).

Duration of preoperative chemotherapy was also found to be associated with TRG. Of the 380 metastases, 282 (74.2%) were resected from 113 patients who received 2-week regimens (short duration: 112 metastases from 50 patients; long duration: 170 from 63 patients), and 98 (25.8%) were resected from 46 patients who received 3-week regimens (short duration: 51 metastases from 19 patients; long duration: 47 metastases from 27 patients). The metastases from patients with long-duration chemotherapy had significantly higher pathologic response rate than those from patients with short-duration chemotherapy (TRG1–2 48.8% and TRG3 37.8% vs. TRG1–2 25.8% and TRG3 38.0%, *P* < 0.001; Fig. 3b).

The serum CEA levels before preoperative chemotherapy were not recorded for 30 patients with 85 metastases in this study. Of the remaining 295 metastases, 226 (76.6%) were from the patients who presented a serum CEA decline after chemotherapy, whereas 69 (23.4%) were from the patients who showed no decrease in the serum CEA level. Moreover, 182 (80.5%) of 226 metastases from the patients with CEA decline were classified as TRG1–2 or TRG3; the metastases from patients who presented CEA decline had significantly higher pathologic response rate than those from patients who did not show serum CEA decline (TRG1–2 46.0% and TRG3 34.5% vs. TRG1–2 18.8% and TRG3 42.0%, *P* < 0.001, Fig. 3c).

An abundance of evidence indicated the association between primary tumor site of metastatic colorectal cancer and radiologic response to chemotherapy. As far as pathologic response was concerned, 67 metastases (17.6%) were from 29 patients with primary tumors on the right side, whereas 313 (82.4%) metastases were from 130 patients with primary tumors on the left side. Metastases from patients with left-side primary tumors had significantly higher pathologic response rate than those from patients with right-side primary tumors (TRG1–2 41.2% and TRG3 38.3% vs. TRG1–2 28.4% and TRG3 35.8%, *P* = 0.008; Fig. 3d).

Different chemotherapy regimens were considered factors that might influence TRG. Of the three groups of chemotherapy regimens, chemotherapy administered with or without targeted therapy did not show a significant difference in the pathologic response rates (TRG1–2 64.2% and TRG3 14.2% vs. TRG1–2 67.4% and TRG3 10.4% vs. TRG1–2 68.2% and TRG3 9.1%, for the non-targeted, bevacizumab-combined, and cetuximab-combined therapy groups, respectively, *P* = 0.442). However, for metastases from the patients treated with cytotoxic chemotherapy alone (non-targeted therapy), the metastases from patients with the oxaliplatin-based chemotherapy showed higher pathologic response rate than those from patients with the irinotecan-based chemotherapy (TRG1–2 44.0% and TRG3 33.7% vs. TRG1–2 13.5% and TRG3 55.8%, *P* = 0.001). For metastases from patients treated with cytotoxic chemotherapy in combination with bevacizumab (bevacizumab-combined therapy), the metastases from patients in the oxaliplatin-based group had higher pathologic response rate than those from patients in the irinotecan-based group, but the difference was not statistically significant (TRG1–2 44.8% and TRG3 41.4% vs. TRG1–2 53.3%, TRG3: 20.0%, *P* = 0.989). Of the 82 metastases from patients treated with chemotherapy in combination with cetuximab (cetuximab-combined therapy), the metastases from patients receiving irinotecan-based chemotherapy presented higher pathologic response rate than those from patients receiving oxaliplatin-based chemotherapy (TRG1–2 43.9% and TRG3 42.4% vs. TRG1–2 12.5% and TRG3 18.8%, *P* < 0.001; Fig. 3e). In metastases from patients who were treated with oxaliplatin-based chemotherapy, those from patients undergoing oxaliplatin/cetuximab therapy presented lower pathologic response rate than those from patients undergoing oxaliplatin-based chemotherapy alone or bevacizumab/oxaliplatin combined therapy (TRG1–2 12.5% and TRG3 18.8% vs. TRG1–2 44.0% and TRG3 33.7% vs. TRG1–2 44.8% and TRG3 41.4%, *P* = 0.001; Fig. 3e). Among metastases from those patients undergoing irinotecan-based chemotherapy, the addition of either bevacizumab or cetuximab led to higher pathologic response rate than chemotherapy alone (TRG1–2 53.3% and TRG3 20.0% or TRG1–2 43.9% and TRG3 42.4% vs. TRG1–2 13.5% and TRG3 55.8%, *P* = 0.048 or < 0.001; Fig. 3e).

## Discussion

In this retrospective cohort study, we showed that the pathologic response after preoperative chemotherapy could predict RFS and OS in a cohort of Chinese colorectal cancer patients with liver metastases who also underwent hepatectomy. Our results supported the results in Western populations [17, 24]. Klinger et al. [24] found that, after liver resection, a favorable TRG score was significantly associated with extended progression-free survival and OS. Moreover, Blazer et al. [17] suggested that pathologic response could be used as a new outcome endpoint for colorectal cancer patients with liver metastases who undergo hepatectomy, because pathologic response strongly predicts postoperative survival.

Blazer et al. [17] also reported that metastases with small size and a preoperative normal CEA level predicted improved pathologic response. Similarly, we found that, in a Chinese patient cohort, metastases with small size were an independent predictor of pathologic response. We also found that a decrease in serum CEA levels after preoperative chemotherapy was another independent predictor of pathologic response. To date, the optimal duration of preoperative chemotherapy remains unknown; to our knowledge, few studies have focused on this issue. Kishi et al. [23] suggested that extended preoperative chemotherapy was associated with an increased risk of liver insufficiency after surgery, but the pathologic response rate was not improved. However, in the present study, we found that long-duration chemotherapy was an independent predictor of pathologic response. This discordance is probably because of different definitions of long-duration chemotherapy. Kishi et al. [23] used a cutoff of 8 cycles to distinguish short-duration from long-duration chemotherapy. However, in the present study, we defined long-duration chemotherapy as longer than 4 cycles for patients who received 2-week regimens and 3 cycles for patients who received 3-week regimens. By this definition, we suggested that a too-short duration of preoperative chemotherapy (< 2.0 months) might not result in satisfactory tumor pathologic regression. In terms of the primary tumor site, this finding was consistent with those of previous studies that found that metastases from the left-side primary tumors had a relatively better response to chemotherapy, as well as to targeted therapy, than metastases from the right-side primary tumors [27–29]. This may due to the genetic heterogeneity of primary tumors on different sides. However, the small sample size, especially for tumors on the right side, indicates that interpretations should be made with caution.

The influence of preoperative chemotherapy in combination with targeted therapy on pathologic response is another source of debate. In the present study, the addition of bevacizumab or cetuximab did not result in a significant difference in pathologic response rates for the whole metastases cohort, but these drugs did have an effect when certain chemotherapy backbones were implemented. These results were in agreement with those of most studies that found that the addition of targeted therapy, including bevacizumab and cetuximab, probably has an effect on the pathologic response of colorectal cancer and liver metastases; however, the effect depended on the chemotherapy backbone [17, 25, 26, 30]. In the present study, for metastases treated with irinotecan-based chemotherapy, the addition of either bevacizumab or cetuximab resulted in higher pathologic response rates than chemotherapy alone. This confirmed the finding of Carrasco et al. [25] that anti-epidermal growth factor receptor agents combined with irinotecan-based regimens were associated with higher pathologic response rates and further supplemented the advantage of bevacizumab/irinotecan-based regimens. In contrast, for metastases treated with oxaliplatin-based chemotherapy, the addition of bevacizumab led to a pathologic response rate similar to that in the chemotherapy-alone subgroup but led to a higher pathologic response rate than that in the cetuximab-combined chemotherapy subgroup. This result was in accordance with the results found in the cetuximab subgroup, in which cetuximab/irinotecan-based chemotherapy resulted in a higher pathologic response rate than the cetuximab/oxaliplatin-based combination. This finding is also supported by previously reported results that cetuximab/oxaliplatin-based regimens were probably not a good choice in terms of pathologic response rate for the treatment of colorectal cancer with liver metastases [25, 26]. However, considering the relatively small sample sizes of the targeted therapy groups in the present study, the results should be interpreted with caution. Additionally, we found that in both the non-targeted therapy and bevacizumab-combined therapy groups, oxaliplatin-based chemotherapy seemed to result in a higher pathologic response rate than irinotecan-based regimens, even though the advantage seen in the bevacizumab subgroup was only a tendency. Carrasco et al. [25] had a similar finding, even though the result did not reach statistical significance in the subgroup that received cytotoxic drugs alone. However, because of the retrospective design and relatively small sample size of these studies, the optimal combination regimen is still unknown. In addition, in the present study, *Kras* status was not recorded for most patients; thus, the association between *Kras* status and pathologic response was not evaluated. Furthermore, in the present study, interobserver variability in terms of the pathologic response and the confounding of resectable/potentially resectable cases may have led to bias. Therefore, prospective randomized trials are needed.

## Conclusions

We found that pathologic response was significantly associated with longer survival after liver resection in colorectal cancer patients with liver metastases. Metastases with a small size, left-side primary tumor, a decrease in the serum CEA level after preoperative chemotherapy, and long-duration chemotherapy might predict high pathologic response rates. However, the optimal combination regimen still needs to be investigated.
